# Benefits of a Multifunctional Sunscreen Formulation Containing Nanoencapsulated Antioxidants in the Skin Protection Against UV Radiation and Blue Light: Clinical and Preclinical Studies

**DOI:** 10.1111/jocd.70282

**Published:** 2025-06-19

**Authors:** Ana Paula Fonseca, Renata Ribon, Lucia Cascais, Gustavo Facchini, Ana Lucia Tabarini Alves Pinheiro, Samara Eberlin, Patricia M. B. G. Maia Campos

**Affiliations:** ^1^ Sallve Comércio de Cosméticos São Paulo SP Brazil; ^2^ Kosmoscience Group Valinhos SP Brazil; ^3^ School of Pharmaceutical Sciences of Ribeirão Preto University of São Paulo São Paulo Brazil

**Keywords:** blue light, efficacy, multifunctional sunscreen, oxidative stress, safety, skin protection

## Abstract

**Background:**

The development of sunscreens with properties beyond photoprotection involves carefully optimizing the blend of ingredients and employing advanced systems to ensure effective delivery through the skin layers.

**Aims:**

The aim of this study was to evaluate the efficacy of a multifunctional photoprotective formulation containing a blend of nanoencapsulated ingredients—ascorbyl palmitate, caffeine, and resveratrol—and the active ingredients niacinamide, hyaluronic acid, bisabolol, tocopherol, and carnosine in skin protection against UV and blue light radiations, by in vitro, ex vivo, and clinical studies.

**Methods:**

A preclinical approach using human explants was performed to evaluate the protective effects of a sunscreen formulation against damage induced by UV and blue light radiation. In addition, a clinical trial was carried out to evaluate skin acceptability, protection from UV‐A and UV‐B, collagen synthesis by fluorescence spectroscopy, and skin hydration.

**Results:**

The results of the preclinical study showed that the proposed formulation prevented UV‐induced reactive oxygen species (ROS) production and stimulated the levels of epidermal opsin‐3 photoreceptors, ensuring melanogenesis control. The clinical study showed that the sunscreen was well‐tolerated by both skin and eyes, improved skin conditions by upregulating collagen synthesis, and moisturizing the skin.

**Conclusion:**

The combination of the active ingredients in the proposed formulation was safe and effective for skin protection against ultraviolet radiation, besides promoting collagen synthesis and opsin‐3 stimulus.

## 1 Introduction

Skin aging is influenced by both genetic and environmental factors, including sunlight and pollutants, also known as the exposome factors. Among these factors, chronic, lifelong exposure to sunlight has an eminent impact on skin aging establishment rate. Importantly, photoaging of human skin is not only caused by ultraviolet radiation but it is also the consequence of exposure to infrared‐A radiation and visible light (VL) [1]. Because of this impact and skin cancer incidence on the population, it is especially important to develop sunscreen formulations, with safety and efficacy guaranteed.

Previous studies showed that the harmful effects of total VL are particularly related to the blue light spectrum range, contributing to the largest share of harmful damage [2]. Among the observed effects are the increase in the production of pro‐melanogenic factors and melanization, using experimental models of cell culture and human skin, reduction in the protein marking of opsin‐3 photoreceptors, which may indicate an imbalance in the phototransduction process and cell signaling affecting the susceptibility of the microenvironment to electromagnetic insult, increased production of free radicals [3] and inflammatory response [4].

Considering there are several factors that contribute to skin photoaging, such as UV radiation and VL, it is very important to develop sunscreen formulations containing an association of active ingredients with antioxidant activity, such as tocopherol, the peptide carnosine, ascorbyl palmitate, caffeine, and resveratrol, as well as hygroscopic properties such as hyaluronic acid (HA), in order to maintain adequate hydration in the *stratum corneum*. Thus, the discovery of mechanisms adjacent to those of protection by absorption, reflection, and dispersion of incident light constitutes a constant challenge for the availability of a photoprotective formulation with multifunctional benefits.

Nanotechnology has a major importance in optimizing the active ingredients in the formulation, ensuring their stability inside the formulation—preventing their oxidation and transformation—and delivering the active ingredient more efficiently in the skin. It means this technology can enable precise delivery of active components to the correct layer of the skin, allowing the combination of different active ingredients without causing incompatibilities with other components in the formula. Nanotechnology also improves the solubilization of the formulation ingredients, providing a more pleasant texture and superior sensory experience compared to using these components without nanocapsules. Lastly, the daily use of the product causes the contact of the formulation with the environment: air (oxygen, water vapor), light, microorganisms, so nanotechnology can protect the active ingredients of the formulation against a variety of aggressions [5].

To prove the efficacy of the formulation, it is important to evaluate the benefits beyond photoprotection. For this, instrumental measurements are important tools for both ex vivo and in vivo methods. Among them, it is possible to mention fluorescence microscopy for cells and tissue morphology and biophysical evaluations, such as the capacitance method to evaluate stratum corneum hydration and fluorescence spectroscopy for collagen synthesis in vivo.

In this context, the objective of this study was to evaluate the efficacy of a multifunctional photoprotective formulation containing a blend of nanoencapsulated ingredients—ascorbyl palmitate, caffeine, and resveratrol—and the active ingredients niacinamide, HA, tocopherol, and carnosine in skin protection against UV and blue light radiations, by in vitro, ex vivo, and clinical studies.

Finally, this study contributes to a better understanding of VL damage on the skin and the importance of the development of multifunctional sunscreens based on nanoencapsulated antioxidants for broad‐spectrum protection.

## 2 Methods

### 2.1 Development of a Sunscreen Formulation Containing Nanoencapsulated Antioxidants

A photoprotective oil/water emulsion was formulated with emulsifiers of glycerine monostearate, polyoxyethylene stearate, and co‐emulsifier composed of citric acid esters of mono‐ and diglycerides in combination with Caprylic/Capric Triglyceride. A blend of organic UV filters was developed and added: butyl methoxydibenzoylmethane, octocrylene, homosalate, ethylhexyl salicylate, and phenylbenzimidazole sulfonic acid. In addition, nanoencapsulated antioxidants, such as caffeine, resveratrol, and vitamin C (ascorbyl palmitate), as well as 2% niacinamide, carnosine, tocopherol, bisabolol, linoleic acid, and ultralow molecular weight HA, were added to this formulation. This formulation will be nominated AHFPS.013A.

The nanoparticles were characterized by specific techniques to ensure their performance and safety. The surface charge was determined by the Zeta Potential, which indicated colloidal stability with values > 30 mV in modulus. The average size of the particles in suspension was measured by dynamic light scattering, revealing nanoparticles smaller than 100 nm, ideal for high topical permeation without reaching the systemic circulation. The morphology and individual size were analyzed by Scanning Electron Microscopy (SEM), which showed particles with a regular and uniform surface, and by transmission electron microscopy, evidencing organized and stable internal structures.

For the stability evaluation, samples were stored in neutral glass containers with tightly sealed lids to prevent the loss of gases or vapor to the environment. Storage temperatures remained constant (5°C, 25°C, and 40°C) throughout a 91‐day period of study, following the guidelines in the Cosmetic Product Stability Guide by the National Health Surveillance Agency (ANVISA). Additionally, during the stability study, the UV filter content in the formulation was monitored in accordance with Brazilian national agency regulations (RDC No. 752, 2022).

### 2.2 Preclinical Trials

#### 2.2.1 Evaluation of Reactive Oxygen Species (ROS) and Opsin‐3 Production in Human Skin Explants

Skin samples were obtained from a single healthy female donor, 35 years old, with skin type II, following an elective abdominal plastic surgery procedure (abdominoplasty). After the surgery, the samples were placed in plastic vials containing 0.9% saline solution and stored under refrigeration for up to 24 h. The skin fragments were then cut into pieces approximately 1.5 cm in size, incubated in culture medium, and treated with the formulation at a dosage of 25–30 mg/cm^2^. The use of human skin samples from elective surgeries in this research was approved by the Ethics Committee of the University São Francisco–SP, under protocol CAAE 82685618.9.0000.5514, opinion no. 2.493.285.

To evaluate the efficacy of the formulation, ex vivo human skin fragments were treated with 25–30 mg/cm^2^ of the formulation for 72 h. Following this, the fragments were exposed to a 100 J/cm^2^ dose of blue light [6] using the Tritan 450 device, with a wavelength of 450 nm, and the AccuPRO Plus XP 4000 from Spectronics Corporation (Westbury, New York, USA). After an additional 24‐h treatment period, the fragments were collected for semi‐quantification of opsin‐3. To evaluate ROS production, the fragments were subjected to a 10 J/cm^2^ dose of UV using UVA devices 400, Sol 500 H1 Filter, and UV Meter from Honle UV America Inc. (Marlborough, MA, USA). The fragments were then incubated for an additional 24 h with AHFPS.013A formulation and subsequently collected for labeling and semi‐quantification of ROS production using the DCFH‐DA probe (2′,7′‐dichlorodihydrofluorescein diacetate; Sigma‐Aldrich, San Luis, MO, USA).

Skin samples were fixed in 4% buffered paraformaldehyde, cryoprotected, and sectioned into 10 μm slices using a Cryostat (Leica CRYOCUT 1800, Germany). The sections were directly placed on silanized slides, washed with phosphate‐buffered saline, and treated with DCFH‐DA [7]. For immunofluorescence analysis, the slides were incubated overnight with a primary anti‐opsin‐3 antibody (Novus Biologicals, Centennial, CO, USA). Afterward, the sections were washed and exposed to an Alexa Fluor 488 secondary antibody (Goat anti‐Rabbit, Thermo Fisher Scientific). DAPI (4′‐6‐Diamidino‐2‐Phenylindole; DNA marker; Sigma‐Aldrich) was used to stain DNA. The slides were mounted with a specialized mounting medium and analyzed using a Fluorescence Microscope (Olympus, Tokyo, Japan) with the cellSens Standard software (2010 Olympus). The analyzed parameter was the fluorescence intensity generated by DCFH‐DA or opsin‐3 antibody labeling. Following image acquisition, the fluorescence intensity was measured using the ImageJ software and expressed in Arbitrary Units (A.U.) [8].

### 2.3 Clinical Trials

#### 2.3.1 Inclusion Criteria

Thirty‐four female participants, aged 46 ± 6 years, with Fitzpatrick skin types II to IV were enrolled in this study. Participants were nonpregnant, nonlactating, and regular users of facial cosmetics. Eligibility criteria included no skin diseases, no ongoing medical treatments, no use of retinoic acid, chemical peels, or similar procedures within 6 months before the study, no significant sun exposure or tanning methods, and no unusual emotional or physical stress. Participants were required to maintain consistent contraceptive methods, dietary habits, and cosmetic routines throughout the study. Any deviation from the established protocol resulted in exclusion from the study. The Ethics Committee of the University São Francisco–SP approved the study under protocol CAAE 47840121.7.0000.5514, opinion no. 4.823.328.

#### 2.3.2 Treatment Protocol

For the real‐use tests, participants were instructed to apply the formulation evenly across the face and neck once daily in the morning, using circular motions until fully absorbed. The study lasted up to 56 days, with periodic evaluations for each parameter under analysis.

Cutaneous and ocular acceptability, along with perceived efficacy, were assessed over 28 days, based on measurements taken at baseline (T0) and after 28 days (T28d). Collagen production was evaluated using spectroscopy at T0, T28d, and after 56 days (T56d) of use.

Hydration kinetics were analyzed at predefined sites on the forearm at T0 and after a single application at 1 (T1h), 4 (T4h), 8 (T8h), 12 (T12h), and 24 (T24h) hours. The contralateral forearm served as an experimental control.

Table 1 summarizes the treatment protocol for each parameter evaluated.

**TABLE 1:** Treatment protocol to assess clinical safety and efficacy for each endpoint evaluated after treatment.

Study	Number of participants	Duration of treatment	Evaluation time	Application site
Cutaneous and ocular acceptability	34	28 days	T0 and T28d	Face
Perceived efficacy	34	28 dias	T0 and T28d	Face
Collagen production	12	56 days	T0, T28d, and T56d	Face
Skin hydration	20	24 h	T0, T1h, T4h, T8h, T12h, and T24h	Forearm

#### 2.3.3 Acceptability Evaluation

The evaluation of cutaneous and ocular acceptability aimed to identify any potential sensations of discomfort, irritation, or allergic reactions caused by the AHFPS.013A formulation under normal usage conditions. Participants were clinically assessed and monitored by both a dermatologist and an ophthalmologist at T0 and T28d during the home‐use phase of the study. The physicians recorded and classified any adverse events reported by the participants, detailing their location, duration, time of onset, frequency, intensity, progression, and any medical treatments prescribed if necessary. Efficacy perception was measured through a questionnaire completed by participants after 28 days of using the AHFPS.013A formulation. Responses were rated on a five‐point scale. The data collected were analyzed to determine the frequency of ratings for each evaluated attribute, including fine wrinkles, dark circles, hydration, firmness, radiance, skin uniformity, and product absorption.

#### 2.3.4 Collagen Production

This study was conducted based on the hypothesis that the AHFPS.013A formulation could stimulate collagen synthesis in the skin, assessed using fluorescence spectroscopy (Fluoromax—Spectrofluorometer 4C‐TCSPC, Horiba Scientific) [9]. In this method, light emitted by a xenon lamp is passed through a monochromator to select a specific wavelength, which is then directed to the skin via a bifurcated optical fiber. The light interacts with the skin and is reflected back through another fiber optic guide, passing through a second monochromator before reaching the photomultiplier cell. The excitation spectra analyzed in this study included a maximum excitation wavelength for cross‐linked collagen at 340 nm and for tryptophan at 295 nm. The efficacy of the investigational product in promoting collagen synthesis was demonstrated by an increase in the *I*_340_/*I*_295_ ratio (I—intensity) after treatment. Spectra were obtained on the hemiface (either right or left) at baseline (T0), after 28 days (T28d) and 56 days (T56d) of home application of the formulation.

#### 2.3.5 Skin Hydration

The assessment of skin hydration was obtained through capacitance measurements using the Corneometer 825 probe coupled to the Multi Probe Adapter, MPA 5 equipment (CKeletronics, Germany). Two evaluation areas were randomly delimited, on the right or left forearm of each research participant, one side being considered control without product application and the other site for application of AHHF‐013A. After the research participants were acclimatized, baseline capacitance measurements were obtained at both delimited sites. Then, 20 μL of the formulation were spread evenly on the respective site, with the aid of a disposable finger. Research participants remained in the laboratory to perform capacitance measurements after 1, 4, 8, 12, and 24 h of product application.

### 2.4 In Vitro and In Vivo Determination of Sun Protection Factor

The in vitro UVA photoprotection parameters, including UVA‐PF, critical wavelength (λc), and the UVA/UVB ratio, were measured following the ISO24443:2012 protocol [10] using spectral transmittance (Labsphere UV‐2000S Ultraviolet Labsphere Halma Company, São Paulo, Brazil) across the 290–400 nm range. This method involves determining the absorbance curves of a thin film of the product applied to a textured substrate (Helioplate, HelioScreen Labs) before and after exposure to a controlled dose of UV radiation (Atlas SUNTEST CPS+, Atlas Material Testing Solutions).

The static sun protection factor (SPF) was assessed according to ISO24444:2010 standards for cosmetic sun protection testing—in vivo determination of the SPF [11]. Ten female participants, aged 45 ± 11 years, with Fitzpatrick skin types II to III were selected for the study. A dose of 2 ± 0.05 mg/cm^2^ of the investigational product was applied to the skin and left to dry for 20 min before UV light exposure (Multiport 601 with a 300 W xenon arc lamp; Solar Light Co Inc). The SPF value was calculated as the ratio of the minimal erythema dose on skin treated with the AHFPS.013A formulation (MEDp) to that on untreated skin (MEDu), using the formula: SPF = MEDp/MEDu.

### 2.5 Statistical Analysis

Preclinical statistical analysis was performed using the ANOVA test to evaluate variations in the results across all groups. To refine and validate the ANOVA findings, the Bonferroni posttest was applied (GraphPad Prism6, Version 6.01, GraphPad Software Inc., La Jolla, CA, USA). A significance level of 5% was adopted. For clinical studies, data analysis and statistical evaluations were conducted using the paired bimodal Student's *t*‐test, with a 95% confidence interval (GraphPad Prism 6.0, GraphPad Software, San Diego, California, USA).

## 3 Results

### 3.1 AHFPS.013A Formulation Protects Skin Against UVA and UVB Radiations

UVA photoprotection in vitro for AHFPS.013A formulation was determined following the protocol described by ISO24443:201. UVA‐PF values were 10.5 ± 0.1, critical wavelength of 378.0 nm (requirement: ≥ 370 nm), UVA‐FP/SPF ratio of 0.35 (requirement: ≥ 0.33). According to ISO24444:2010 Cosmetics—Sun protection test methods—In vivo determination of the SPF, used to determine the SPF, it was possible to conclude that the formulation presented an average static SPF equal to 32.9. The study was carried out considering the SPF of 30.

### 3.2 AHFPS.013A Formulation Prevents ROS Formation

**FIGURE 1:**
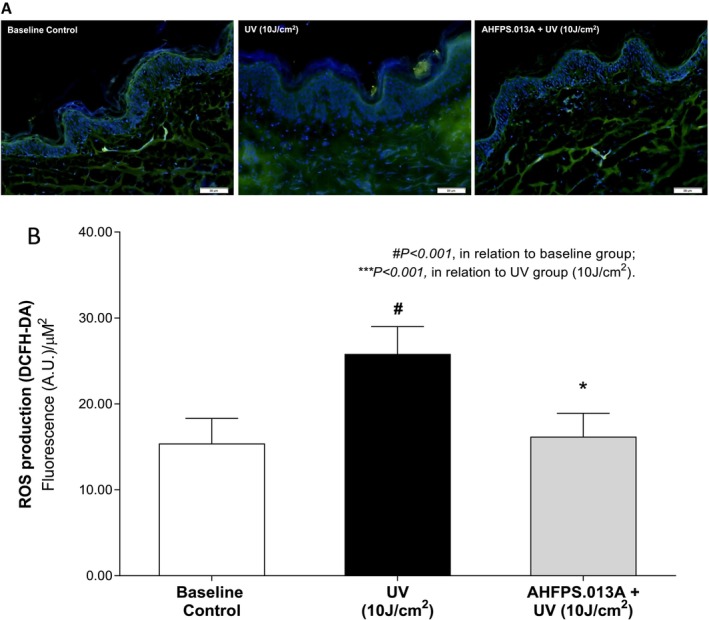
UV‐induced reactive oxygen species (ROS) production is downregulated by treatment with the sunscreen formulation. (A) Microscopic evaluation of ROS labeled in green by DCFH‐DA probe in epidermal and dermal compartments (blue labelling represents cell nucleus) in human skin fragments treated with AHFPS.13A and submitted to UV radiation (10 J/cm^2^). Reference bar corresponds to 50 μm. (B) Semi‐quantification of fluorescence intensity (Arbitrary Units—A.U.) of ROS production. Data represents the mean ± standard deviation of 12 experimental areas (ANOVA, Bonferroni).

Human skin explants were treated with AHFPS.013A (25–30 mg/cm^2^) and exposed to 10 J/cm^2^ of UV radiation. After 24 h, ROS staining was carried out using the DCFH‐DA probe directly on histological sections. Fluorescence is revealed in green by the oxidation of the DCFH‐DA probe in the epidermal and dermal compartments, and blue labeling represents the cell nucleus (Figure 1A). UV radiation exposure resulted in a significant increase in ROS production when compared to the baseline control, leading to the activation of oxidative stress. Conversely, AHFPS.013A showed a protective effect on skin explant cell cultures, reducing ROSmit labeling compared to skin exposed solely to UV radiation. In addition, Figure 1B showed the results of the semi‐quantification of ROS staining/production obtained from the analysis of microscopic images. UV radiation induced a 67.2% increase in the ROS fluorescence over the baseline control, and the AHFPS.013A‐treated group promoted a 37.3% reduction compared to the only UV‐irradiated group (*p* < 0.001).

### 3.3 AHFPS.013A Formulation Favors the Availability of Opsin‐3 Photoreceptors

**FIGURE 2:**
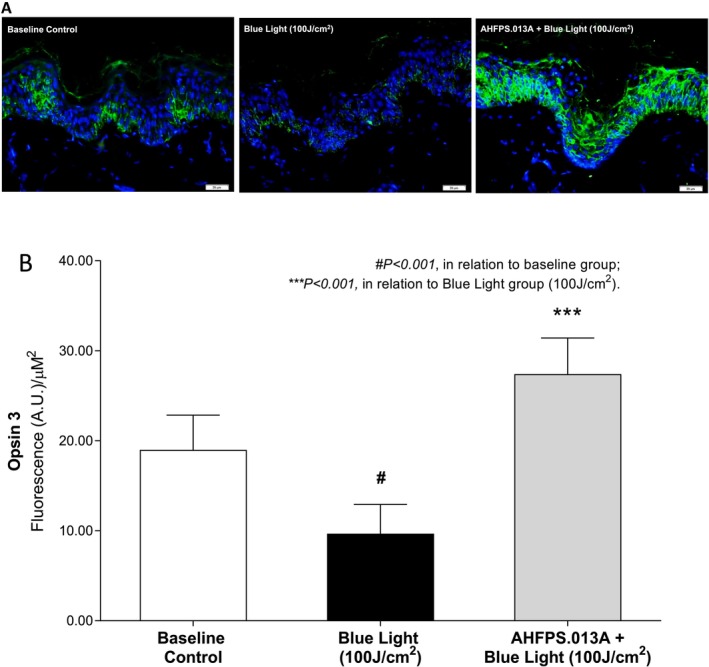
Downregulation of Opsin‐3 induced by 100 J/cm^2^ of Blue Light is reversed by pretreatment with sunscreen formulation (AHFPS.013A). (A) Microscopic immunostaining of Opsin‐3 (green) in epidermal compartment (blue labelling represents cell nucleus) in human skin fragments treated with AHFPS.13A and submitted to Blue Light (100 J/cm^2^). Reference bar corresponds to 20 μm. (B) Semi‐quantification of fluorescence intensity (Arbitrary Units—A.U.) of Opsin‐3 staining. Data represents the mean ± standard deviation of 12 experimental areas (ANOVA, Bonferroni).

Human skin explants were treated with AHFPS.013A at a ratio of 25–30 mg/cm^2^ and exposed to a 100 J/cm^2^ dose of blue light. After 24 h, the immunostaining and semi‐quantification of opsin‐3 was carried out on histological sections. Immunofluorescence for opsin‐3 is revealed in green in the epidermal compartment, and blue labeling represents the cell nucleus (Figure 2A). As expected, blue light showed reduced staining for opsin‐3 compared to non‐photoexposed fragments. The fragments treated with AHFPS.013A demonstrated an increase in the bioavailability of opsin‐3 when compared to the group stimulated with blue light. The semi‐quantification of these results revealed that photoexposure to blue light promoted a 49.04% reduction in the immunostaining of opsin‐3 (*p* < 0.001) when compared to the baseline control. On the other hand, the group treated with AHFPS.013A showed a significant increase of 183.20% (*p* < 0.001) in the intensity of the opsin‐3 labeling when compared with the group only irradiated with blue light.

### 3.4 Effect of AHFPS.013A on Collagen Production and Skin Firmness

**FIGURE 3:**
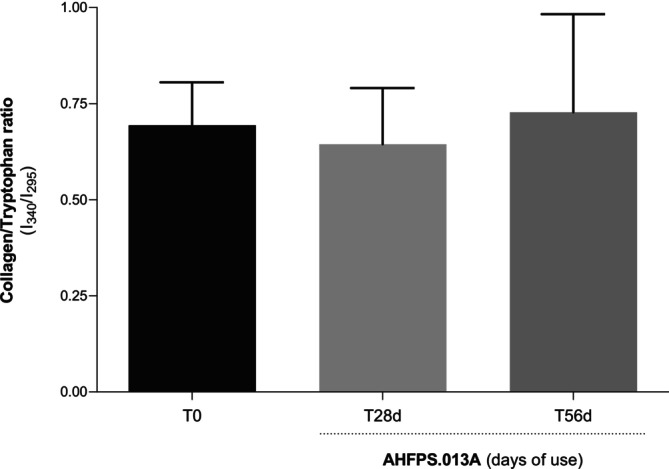
Mean values of the ratio between collagen and tryptophan intensity (*I*_340_/*I*_295_) obtained before and after 28 and 56 days of home use of AHFPS013A. Data represent the mean ± standard deviation (*n* = 12; Student *t*‐test).

Similar outcomes were observed in a clinical trial to assess increased collagen synthesis in twelve research participants by noninvasive diffuse reflectance spectroscopy. Figure 3 illustrates the mean values of the ratio between collagen and tryptophan intensity (*I*_340_/*I*_295_) obtained before and after 28, 56, and 84 days of home use of AHFPS.013A. According to the results obtained in the statistical analysis (Student's test), there was no significant increase in the values of *I*_340_/*I*_295_ after 28 and 56 days of daily use, even despite a slight increase after 56 days of treatment. However, there was a significant (*p* < 0.05) increase in the values of *I*_340_/*I*_295_ after 84 days of daily use (Figure 3). Still associated with this outcome, 85.3% of research participants noticed a reduction in the appearance of expression lines, and 94.1% noticed that the skin became firmer, after 28 days of home use of AHFPS.013A.

### 3.5 Effect of AHFPS.013A on Skin Hydration

**FIGURE 4:**
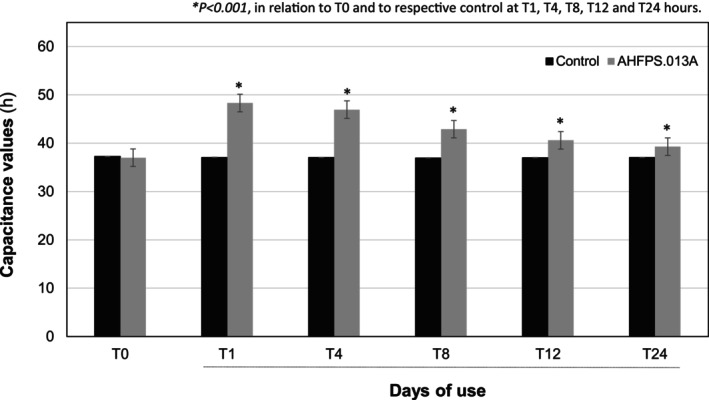
Capacitance values obtained in study subjects before (baseline values‐T0) and after 1 (T1), 4(T4), 8 (T8), 12 (T12), and 24 (T24) hours of the application of AHFPS.013A formulation. Data represents the mean ± standard deviation (*n* = 20; paired bimodal Student *t*‐test).

Figure 4 shows the average capacitance values (h) recorded throughout the study following the application of AHFPS.013A, measured using the corneometry method. The results revealed a significant increase (*p* < 0.001) in capacitance values at 1, 4, 8, 12, and 24 h after AHFPS.013A application, suggesting that the product effectively enhanced skin hydration. These increases were deemed significant when comparing the treated areas with both the initial baseline control (T0) and the corresponding controls at T1, T4, T8, T12, and T24 h, showing an improvement of up to 30.42%. Additionally, 94.1% of the participants reported that the product kept their skin hydrated after 28 days.

### 3.6 AHFPS.013A Demonstrated Positive Ocular and Cutaneous Tolerability

A total of 34 research participants were clinically assessed by both a dermatologist and an ophthalmologist at baseline and after 28 days of at‐home use of AHFPS.013A. Following 28 days of product use, clinical evaluations confirmed that 100% of participants exhibited no adverse reactions, signs, or symptoms of skin discomfort, nor did they report any irritative or allergenic reactions or sensations of discomfort associated with the product. Similarly, 100% of participants experienced no adverse reactions, signs, or symptoms of ocular discomfort, and did not report any irritative or allergenic reactions or sensations of ocular discomfort resulting from the use of AHFPS.013A. Throughout the study, no adverse events were reported or documented.

## 4 Discussion

The demand for effective skincare cosmetic products that not only provide photoprotection but also address specific skin concerns is increasing. Consumers are seeking products that offer both skin protection and enhanced aesthetics, contributing to a more youthful and radiant appearance. To meet these needs, the cosmetic industry incorporates active ingredients with multifunctional properties, including antioxidant, antiaging, photoprotective, and anti‐inflammatory effects [12]. These ingredients enhance the efficacy of photoprotective products, offering greater skin protection and improving overall skin health.

In line with this reasoning and focusing on the anti‐aging properties that can be attributed to the photoprotector, we highlight the protection against the harmful effects of the VL spectrum, particularly the blue light range (400–500 nm). Tropical countries receive a higher amount of solar radiation throughout the year, which can cause a bigger impact on skin aging rate, with these age signs appearing earlier in life and more abundantly, such as dark spots formation, especially in phototype IV people—a large population in tropical countries. Blue light penetrates skin more extensively than UVA and UVB rays, resulting in overproduction of ROS, DNA damage, breaking of extracellular matrix proteins, pigmentation changes, and evidencing the skin aging process [6, 8].

Studies have highlighted the potential effects of blue light exposure on the skin, revealing its ability to stimulate melanocytes and contribute to pigmentation concerns like melasma and age spots. Although the mechanisms behind UV‐induced melanogenesis are well documented, the processes associated with blue light‐induced skin hyperpigmentation remain unexplored. However, a study proposes that the mechanism underlying hyperpigmentation caused by blue light differs from that of UV light. It suggests that the pigmentation triggered by blue light involves opsins, a group of photosensitive proteins located in the epidermis [13].

Opsins are photosensitive components and belong to the superfamily of G protein‐coupled hepta‐helical receptors. These cell surface receptors are activated by a wide variety of stimuli and drive signaling across biological membranes [13]. Haltaufderhyde et al. [13] demonstrated that the mRNA and protein opsin 3 are expressed at higher levels than other opsins in the two main types of human epidermal cells, melanocytes and keratinocytes. Activation of the opsin‐3 receptor by blue light triggers a process of perinuclear aggregation of this receptor. This formation of aggregates has important consequences, among them, the direct stimulation of the production of ROS and reduction of the availability of free opsin‐3 receptors. This results in a rapid increase in ROS contributing to the onset of oxidative stress. Furthermore, the reduction or formation of opsin‐3 aggregates can induce the exacerbated production of melanin and culminate in unsightly hyperchromias [14].

In this study, we evaluated the protective effects of a sunscreen formulation—UVA‐PF 10.5 ± 0.1, critical wavelength of 378.0 nm, SPF 32.9, UVA‐FP/SPF ratio of 0.35, on human skin explants subjected to radiation with blue light. The results showed that exposure of the fragments to a dose of 100 J/cm^2^ of blue light resulted in reduced staining for opsin‐3 compared to non‐photoexposed fragments. This event occurred due to the formation of opsin‐3 aggregates induced by radiation and the consequent reduction in the availability of these photoreceptors, which was confirmed by the significant reduction in detection by the specific antibody. According to Ozdeslik et al. [8] the reduction of opsin‐3 is associated with an increase in melanin in human melanocytes through activation of the melanocortin‐1 receptor (MC1R), indicating an important role for opsin‐3 as a regulator of melanogenesis.

Another adjacent mechanism presented by sunscreen formulation was the antioxidant activity, demonstrated in this study through the quantification of ROS in human skin explants exposed to UV radiation. In this experiment, photoexposure to UV radiation resulted in a significant increase in ROS production, which was practically inhibited by treating the fragments with the photoprotective formulation.

This result corroborates the composition of the product that contains ingredients with known anti‐aging properties. Among them are resveratrol, caffeine, ascorbyl palmitate, alpha‐tocopherol, and carnosine, important antioxidants that act in the natural defense of skin, playing a fundamental role in free radical scavenging and collagen biosynthesis [15, 16, 17, 18, 19, 20, 21, 22, 23].

Resveratrol, a polyphenol compound found in *Vitis vinifera* , has a wide range of beneficial biological actions, including antioxidant action by mechanisms involving the modulation of nuclear factor erythroid 2‐related factor 2 (Nrf2) and sirtuin 1. In addition to its potential antioxidant action, tocopherol exerts a fundamental role in protecting DNA by preventing the formation of pyrimidine photoproducts, anti‐inflammatory effects through inhibition of eicosanoid and cyclooxygenase‐2 secretion, suppression of pro‐inflammatory signaling pathways NF‐κB and STAT‐3 [16]. Carnosine complements these actions, suppressing telomere shortening, preventing advanced glycation end products formation, preserving sirtuin signaling, and rejuvenating senescent cells [17].

Clinical responses of AHFPS.013A formulation in collagen production were also evaluated using a non‐invasive method based on fluorescence spectroscopy. The characterization of skin fluorescence in terms of native fluorophores, such as NADH, collagen, elastin, tryptophan, and porfirins, has been proposed as a way of differentiating healthy skin from diseased and intrinsic aging from photoaged skin [24]. Therefore, this method constitutes an effective way to assess the amount of dermal collagen. In our study, research participants were instructed to use AHFPS.013A formulation for 84 consecutive days, and the increase of collagen was evaluated by calculating the ratio between the excitation spectra obtained for collagen and tryptophan (*I*_340_/*I*_295_). The treatment efficacy was not statistically evidenced by the increase in the *I*_340_/*I*_295_ ratio after 28 or 56 days of use; however, there was a significant (*p* < 0.05) increase after 84 days of daily use. This effect on collagen production, associated with the perception of improvement in the appearance of fine wrinkles (85.3%) and skin firmness (94.1%) declared by the participants, suggests that the effect of increasing collagen production can be intensified with prolonged treatment of AHFPS.013A. Moreover, it highlights the importance of daily use of the investigational product in order to promote benefits on skin. This observed effect can be attributed to several components of the AHFPS.013A formulation, especially resveratrol, vitamin C, caffeine, carnosine, and HA.

The study also investigated the improvement of periorbital conditions, a persistent and aesthetically challenging sign of aging. Often referred to as “dark circles,” alterations in the color and shape of the eyelids can manifest as hyperpigmentation or hyperchromia, under‐eye bags, eyelid edema, and the early onset of fine wrinkles [24]. It is well established that the skin surrounding the periorbital region is particularly vulnerable to the detrimental effects of aging. Even individuals with otherwise healthy skin often experience notable changes in this area. In this regard, 88.2% of survey participants self‐reported a reduction in the appearance of dark circles under the eyes. According to the results reported by the research participants, AHFPS.013A formulation favored a reduction in the appearance of fine lines and the tone of dark circles, suggesting its use as an eyecare as well.

Instrumental evaluation methods, such as Mexameter or image analysis, would be a well‐suited techniques in this paper; however, it was chosen to use a validated self‐assessment questionnaire with subjects, considering that the subjective perception of aesthetic improvement is a relevant endpoint in cosmetic studies, particularly when the focus is on consumer satisfaction and perceived efficacy. This approach is widely accepted in clinical studies with cosmetic products in accordance with international guidelines such as those from Cosmetics Europe and the principles outlined in Regulation (EU) No 655/2013 on the justification of cosmetic product claims.

The formulation studied also contains linoleic acid and oleic acid that complement the benefits of this formulation through mechanisms that involve the maintenance of skin barrier function. These unsaturated fatty acids enter various metabolic pathways such as phospholipid synthesis in the basal layer of the epidermis, where the cells are proliferating. During the differentiation process, the catabolism of phospholipids favors keratinocytes to produce neutral lipids, such as triglycerides and ceramides, which form the stratum corneum lipid matrix [25]. These properties of the proposed formulation were confirmed in this study through the hydration kinetics produced after a single application of the product in the forearm of the research participants. Capacitance measurements were taken up to 24 h after application and indicated that the product provided a significant increase in skin hydration over the time evaluated, reaching a 30.42% increase compared to the untreated site. It is important to note that 100% of the panel showed an increase in the capacitance indexes after 1, 4, and 8 h of application, 90% after 12 h, and 75% after 24 h, compared to the respective control site.

Assessing the skin surface water content is crucial when examining the efficacy of cosmetic products designed for this specific purpose. Among the primary methods for determining the hydration of the outermost layer of the skin and quantifying its moisturizing impact, the capacitance method stands out as widely utilized. Skin hydration provided by the application of a moisturizing product is evidenced by the increase in the capacitance value generated between the base of the Corneometer probe and the skin. The higher the capacitance value, the greater the amount of water on the skin surface and, therefore, the greater its hydration level.

Besides unsaturated fatty acids, HA also present in the formulation and responds by moisturizing, protecting the skin and providing anti‐aging properties. It has been acknowledged for its ability to retain and replenish moisture in the skin, resulting in a softer, smoother, and radiant appearance. Skin hydration also leads to delaying the formation of wrinkles and improves deep fine lines and already developed wrinkles that usually appear with age. In addition to the antioxidant effects, HA also promotes cell regeneration and stimulates collagen production, showing the multifaceted mechanism of this macromolecule [23].

The balance between skin health and damage is seen today as a holistic approach, considering intrinsic and extrinsic factors, such as genome (genes, age/gender), exposure: external (sun, pollution, climate), lifestyle factors (sleep, stress, exercise, nutrition, skin care routine), as well as the role of our skin microbiome [24]. Skin aging does not result from an isolated mechanism, but from a set of persistent deleterious effects that eventually compromise tissue homeostasis. Age‐related changes result in cumulative detrimental effects characterized by abnormal ECM organization, pigmentary changes, loss of subcutaneous fat, hair graying, minor hair density, decreased sebaceous gland function, and low‐grade chronic inflammation [24]. Chronologically aged skin is characterized by laxity and some exaggerated expression lines, whereas extrinsic aging signs include dryness, rhytids, irregular pigmentation, loss of elasticity, telangiectasias, and areas of purpura [25].

In summary, the proposed photoprotective formulation showed a multifunctional performance to neutralize the various molecular alterations underlying the cellular dysfunction related to aging. Thus, in this study we present a sunscreen formulation with essential ingredients to contain clinical signs of skin aging. The proposed formulation was well‐tolerated by both the skin and eyes, improved skin appearance by upregulating collagen synthesis, and moisturized the skin. Besides UVA and UVB photoprotection, the formulation prevented UV‐induced ROS production and stimulated the availability of epidermal opsin‐3 photoreceptors, reduced by exposure to blue light, ensuring a better melanogenesis control. These benefits were achieved through the combination of effective ingredients and a nanocapsule‐based delivery system that improved solubility, safety, and efficacy of the formulations in the skin.

## Author Contributions

Ana Paula Fonseca and Samara Eberlin were responsible for the study conceptualization. Ana Paula Fonseca, Renata Ribon, Gustavo Facchini, Samara Eberlin Ana Lúcia were responsible for methodology. Ana Paula Fonseca, Samara Eberlin, Lucia, and Patricia Maia Campos were responsible of formal analysis. Ana Paula Fonseca, Renata Ribon, Patricia Maia Campos and Samara Eberlin were responsible of writing original draft preparation. Ana Paula Fonseca and Patrícia Maia Campos were responsible of review and editing.

## Ethics Statement

The authors have nothing to report.

## Conflicts of Interest

Ana Paula Fonseca, Renata Ribon and Lucia Cascais are employees of Sallve Cosméticos. Samara Eberlin, Gustavo Facchini and Ana Lucia Tabarini Alves Pinheiro are employees of Kosmoscience Research Institute. Patricia M. B. G. Maia Campos is Professor at Universidade de São Paulo. No conflicts of interest is noted.

## Data Availability

The authors have nothing to report.
